# Genetic Diversity and Antimicrobial Drug Resistance of Serotype VI Group B *Streptococcus,* Canada

**DOI:** 10.3201/eid2410.171711

**Published:** 2018-10

**Authors:** Alefiya Neemuchwala, Sarah Teatero, Lindsay Liang, Irene Martin, Walter Demzcuk, Allison McGeer, Nahuel Fittipaldi

**Affiliations:** Public Health Ontario Laboratory, Toronto, Ontario, Canada (A. Neemuchwala, S. Teatero, L. Liang, N. Fittipaldi);; National Microbiology Laboratory, Public Health Agency of Canada, Winnipeg, Manitoba, Canada (I. Martin, W. Demzcuk);; University of Toronto, Toronto (A. McGeer, N. Fittipaldi);; Mount Sinai Hospital, Toronto (A. McGeer)

**Keywords:** Streptococcus group B, Streptococcus agalactiae, genomics, genetic diversity, drug resistance, microbial, bacteria, Canada, antimicrobial resistance

## Abstract

Two genetically dissimilar sequence type 1 clades dominate the serotype VI group B *Streptococcus* population of strains causing invasive disease in Canada. Isolates of this rare serotype, recovered mainly from adult patients, were all susceptible to penicillin and vancomycin. However, we observed resistance to erythromycin and clindamycin.

Serotype VI group B *Streptococcus* (GBS), which is common in Japan (prevalence rates 16%–40%) and has recently emerged in Malaysia and Taiwan, remains rare in Europe and North America (*1*–*4*). However, invasive serotype VI infections have been noticed in Alberta and Ontario, Canada (*5*,*6*), and unpublished surveillance data for Canada (National Microbiology Laboratory, https://www.canada.ca/en/public-health/services/publications/drugs-health-products/national-laboratory-surveillance-invasive-streptococcal-disease-canada-annual-summary-2015.html) show low frequency (1.2%–4.1%) but sustained isolation of this GBS serotype in recent years. Here, we characterize a collection of 26 invasive serotype VI GBS strains recovered by passive surveillance in central Canada during 2010–2014 (Technical Appendix 1). Two isolates came from early onset disease (patients age 0–6 days) and 1 from late-onset disease (patients age 7–90 days). Twenty-two isolates came from adult patients (9 age 18–60 years and 13 age >60 years, a distribution similar to that reported for adult patients with serotype V or serotype IV invasive disease in Canada [*5*,*6*]). Patient age was not available for 1 isolate.

We sequenced the genomes of all isolates using Illumina technology (Illumina, San Diego, CA, USA; National Center for Biotechnology Information BioProject PRJNA420560) and performed in silico multilocus sequence typing. Isolates belonged to sequence types (STs) ST889 (n = 1), ST297 (n = 1), ST14 (n = 2), and ST1 (n = 22) (Technical Appendix 1). ST297, ST14, and ST1 are members of clonal complex (CC) 1; most serotype IV and V isolates responsible for adult disease in Canada also belong to CC1 (*5*–*7*). However, genome-wide, single-nucleotide polymorphism (SNP)–based phylogenetic analysis showed that CC1 isolates of these 3 serotypes are genetically dissimilar (Technical Appendix 2 Figure 1; genome-wide SNPs were identified relative to the genome of GBS-M002, a serotype VI isolate from Taiwan [GenBank accession no. CP013908.1]). Antimicrobial drug resistance among serotype VI isolates was, overall, similar to that described among serotype IV and V isolates causing adult invasive disease in Canada (*5*,*8*) (MICs for penicillin, erythromycin, clindamycin, tetracycline, and vancomycin were determined using the agar dilution method or Etest according to Clinical and Laboratory Standards Institute guidelines [*9*]). All serotype VI isolates were susceptible to penicillin and vancomycin (Technical Appendix 1). Resistance to erythromycin was found in 10 (38%) invasive isolates, resistance to clindamycin in 9 (35%), and resistance to tetracycline in 8 (31%) (Technical Appendix 1). All lincosamide- and macrolide-resistant strains possessed gene *ermB*; 1 isolate had genes *mefA* and *msrD*. Genes *tetS*, *tetM*, and *tetO* were associated with observed resistance to tetracycline (Technical Appendix 1).

Most (n = 22) ST1 isolates in our collection had a pilus island (PI) profile consisting of PI-1 containing the recently described PI-1 backbone protein subunit BP-1b (*10*) (BP1b-PI-1), in combination with PI-2a (Technical Appendix 1). One ST1 isolate (NGBS1605) possessed the traditional PI-1 and PI-2a (Technical Appendix 1). The ST14 isolates had BP1b-PI-1 and PI-2b. The ST889 isolate possessed only PI-2a (Technical Appendix 1). We found differences among isolates in genes encoding α-like proteins (Alps): the ST297 isolate and most ST1 strains had gene *bca* encoding α-C protein. ST1 isolates NGBS543 and NGBS1605 possessed gene *alp3*, encoding Alp3. The ST14 and ST889 isolates possessed gene *alp1*, encoding Alp1 (or epsilon) protein (Technical Appendix 1).

We next examined the extent of genetic diversity among the numerically dominant group of serotype VI ST1 organisms. For comparative purposes, genome data for 3 additional serotype VI strains were included (French strain CCH330, SRA accession no. ERX298473; Malaysian strain PR06, GenBank accession no. AOSD00000000.1; and 1 temporally matched serotype VI isolate recovered from a colonized pregnant woman in Canada; Technical Appendix 1). Recombination was the main driver of genetic diversity among serotype VI ST1 organisms. Most (n = 16) ST1 isolates clustered closely with Malaysia strain PR06 (Technical Appendix 2 Figure 2). This clade (arbitrarily named the Malaysian clade) included most ST1 isolates with resistance to erythromycin and clindamycin. Recombination in a region of ≈200 kbp containing the genes encoding the 2-component virulence regulator CsrRS differentiated the Malaysian clade from a second clade formed by 5 Canadian isolates and the French and Taiwanese ST1 isolates (arbitrarily named the Taiwanese clade) (Technical Appendix 2 Figure 2). Recombination also explains the aforementioned differences in Alp- and pilus subunit–encoding genes among serotype VI ST1 strains. Isolates NGBS543 and NGBS1605 differed from other ST1 isolates by recombination in a region spanning 107 and 89 kbp, respectively, containing Alp-encoding genes. These 2 isolates also differed between themselves by recombination in the PI-1 locus (Technical Appendix 2 Figure 2).

Global travel and migration are known contributors to the emergence of bacterial clones in new geographies (*11*). Serotype VI GBS infections have emerged in Malaysia and Taiwan (*3*,*4*). The population of serotype VI GBS isolates in Canada is dominated by 2 ST1 clades, each closely related genetically to the Malaysian or Taiwanese isolates. Although it is tempting to speculate that these 2 ST1 genotypes were introduced into Canada from overseas, the speculation cannot be fully supported by our current limited dataset. Continued monitoring for serotype VI GBS infections is warranted.

**Technical Appendix 1.** Characteristics of serotype VI group B *Streptoccoccus* isolates used in this study.

**Technical Appendix 2.** Figures describing phylogenetic relationships among serotype VI group B *Streptococcus* strains.
