# Supplementary material for: Genetic Diversity and Antimicrobial Drug Resistance of Serotype VI Group B Streptococcus, Canada
Source: Emerg Infect Dis. 2018 Oct;24(10):1941–2. doi: 10.3201/eid2410.171711 (PMC6154151; doi:10.3201/eid2410.171711)
Supplement: Supplementary file 2 — Technical Appendix 2. Figures describing phylogenetic relationships among serotype VI group B Streptococcus strains. [file 17-1711-Techapp-s2.pdf]

Article DOI: https://doi.org/10.3201/eid2410.171711

Genetic Diversity and Antimicrobial Drug Resistance of Serotype VI Group B *Streptococcus,* Canada

Technical Appendix 2

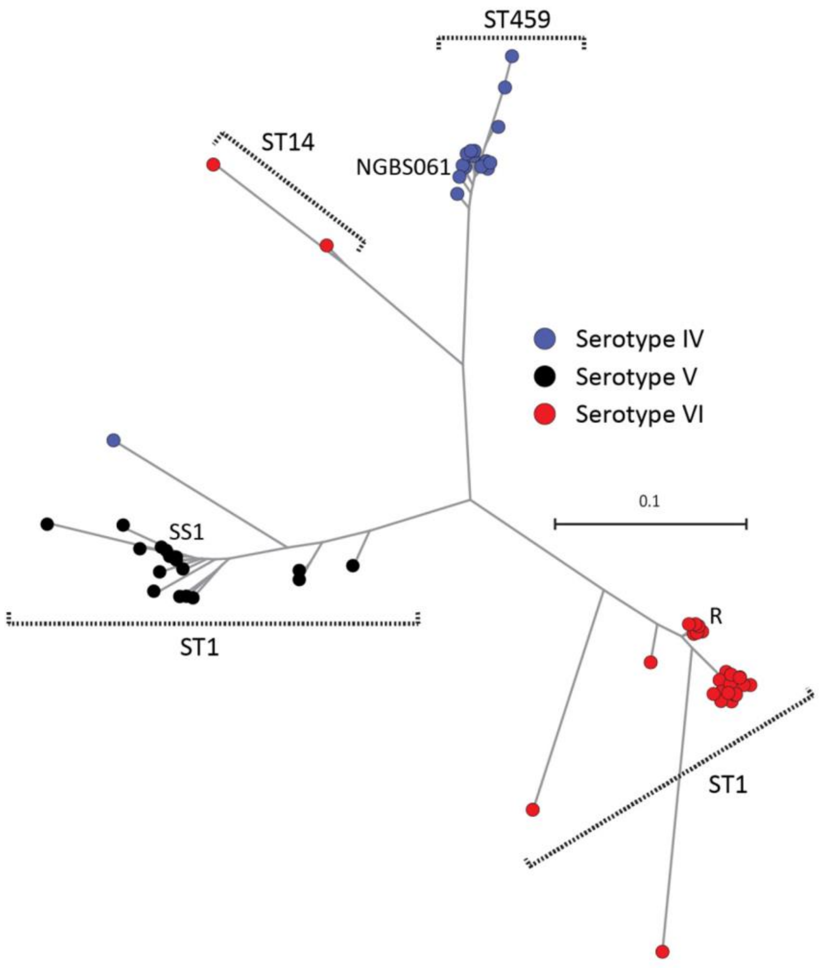

Technical Appendix 2 Figure 1. Inferred phylogenetic relationships between clonal complex 1 group B *Streptococcus* (GBS) strains of serotype VI analyzed in this study, and selected clonal complex 1 GBS strains of serotypes IV and V. The neighbor-joining phylogenetic tree (1,000 bootstrap replications) was constructed using 22,571 nonredundant biallelic single-nucleotide polymorphism (SNP) loci identified in the genomes of the strains relative to the core genome of the reference strain GBS-M002 (labeled R,

GenBank accession no. CP013908.1). The analysis shows that these CC1 isolates are genetically dissimilar. The serotype IV strains used (SRA accession numbers) are NGBS1098 (SRS1211973), NGBS049 (SRS586361), NGBS058 (SRS586362), NGBS061 (SRS586363), NGBS191 (SRS586371), NGBS258 (SRS586374), NGBS410 (SRS586379), NGBS400 (SRS586380), NGBS493 (SRS586383), NGBS507 (SRS586384), NGBS525 (SRS586386), NGBS024 (SRS586396), NGBS686 (SRS960463), NGBS680 (SRS960464), NGBS698 (SRS960467), NGBS700 (SRS960483). The serotype V strains used (SRA accession numbers) are NGBS571 (SRS837765), NGBS561 (SRS837766), NGBS558 (SRS837768), NGBS553 (SRS837769), NGBS519 (SRS837774), NGBS513 (SRS837775), NGBS499 (SRS837776), NGBS494 (SRS837778), NGBS492 (SRS837779), NGBS462 (SRS837780), NGBS444 (SRS837781), NGBS434 (SRS837783), NGBS425 (SRS837784), NGBS418 (SRS837785), NGBS380 (SRS837788), NGBS372 (SRS837789), SS1 (SRS837702).

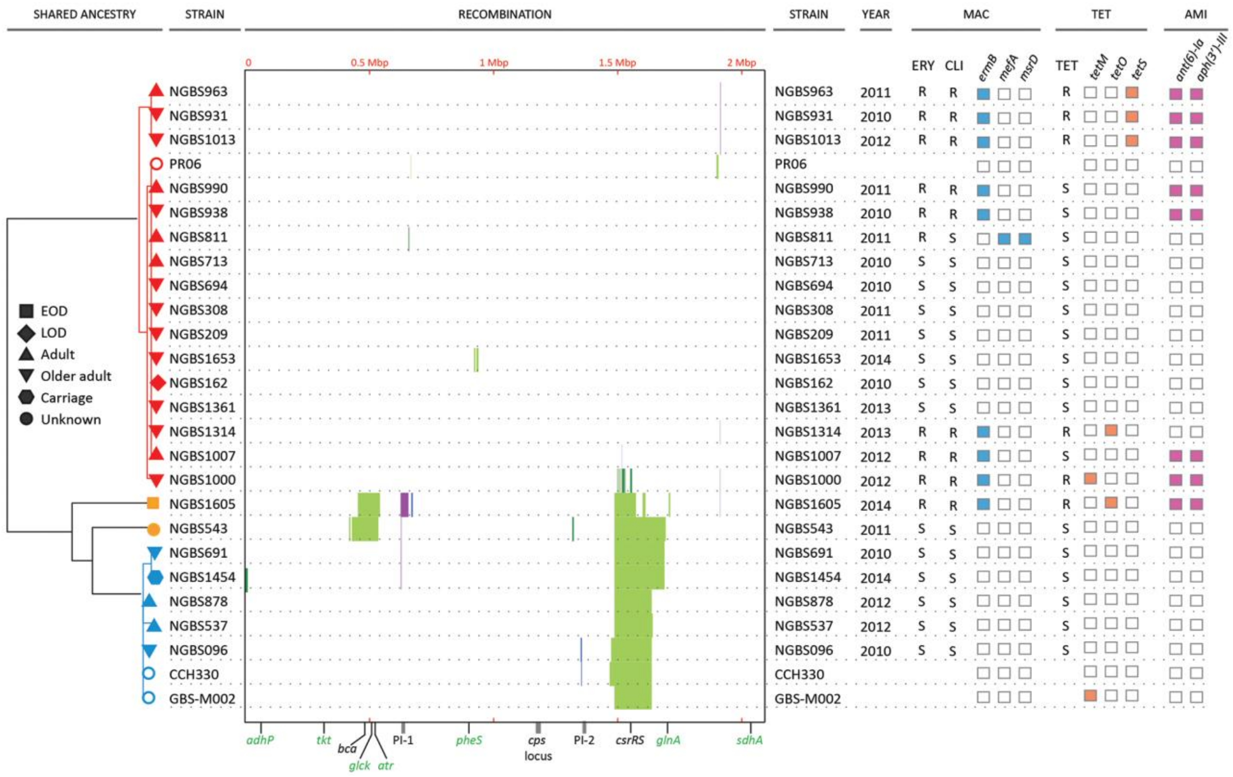

Technical Appendix 1 Figure 2. Phylogenetic relationships, recombination, and antimicrobial resistance among sequence type 1 (ST1) serotype VI group B *Streptococcus* strains. The left panel shows a proportion of shared ancestry tree built with BratNextGen (http://www.helsinki.fi/bsg/software/BRAT-NextGen/) and based on 6,802 nonredundant single-nucleotide polymorphism loci identified in the genomes of 23 serotype VI ST1 GBS isolates from Canada (22 invasive and 1 colonizing isolates) and 1 additional serotype VI ST1 strain each from Malaysia (strain PR06) and France (strain CCH330), relative

to the genome of strain GBS-M002 (a serotype VI isolate from Taiwan). Three different clades are indicated by different colors. Most ST1 strains clustered tightly with Malaysian strain PR06, defining an arbitrarily named Malaysian clade (in red). A second, arbitrarily named Taiwanese clade, is shown in blue. Two other strains form a unique cluster, shown in orange. The center boxed panel depicts results of Bayesian analysis of recombination. The colored bars denote statistically significant recombination events identified in each strain relative to the genome of strain GBS-M002. The coloring of the bars at a specific genomic location reflects the clustering of the recombination events into groups, and is unrelated to other bars at distant genomic locations. The position of the 7 genes used in the GBS multilocus sequence typing scheme (*adhP*, *tkt*, *glcK*, *atr*, *pheS*, *glnA*, and *sdhA*, in green), as well as the positions of the capsule (*cps*) locus, the pilus island (PI)-1 and PI-2 loci, and the genes encoding the α-C protein and the 2-component virulence regulator CsrRS, are provided for reference. The right panel shows the presence of genes encoding antimicrobial resistance (indicated by the colored boxes), as well as phenotypic resistance results for macrolides and tetracycline. Age groups are depicted by the different geometric shapes. EOD: early-onset disease (patient 0–6 days of age); LOD: late-onset disease (patient 7–90 days of age); adult (18–59 years of age); older adult (≤60 years of age).
